# Mental Health among Geriatric Healthcare Workers in Italy during the COVID-19 Pandemic: Results from a National Survey

**DOI:** 10.1007/s12603-023-1958-1

**Published:** 2023-08-11

**Authors:** Alice Margherita Ornago, E. Pinardi, A. Zucchelli, C. Trevisan, M. Volterrani, S. Cacciatore, C. Ceolin, F. Landi, M. Trabucchi, D. De Leo, A. Bianchetti, G. Bellelli, YES group

**Affiliations:** 1 School of Medicine and Surgery, University of Milano-Bicocca, Piazza dell'Ateneo Nuovo, 1, Milan, Italy; 2 Department of Clinical and Experimental Sciences, University of Brescia, Brescia, Italy; 3 Department of Medical Science, University of Ferrara, Ferrara, Italy; 4 Department of Psychology, Università Cattolica del Sacro Cuore, Brescia, Italy; 5 Department of Geriatrics and Orthopedics, Università Cattolica del Sacro Cuore, Rome, Italy; 6 Department of Medicine, University of Padua, Padua, Italy; 7 Fondazione Policlinico Universitario “Agostino Gemelli” IRCCS, Rome, Italy; 8 Italian Association of Psychogeriatric (Associazione Italiana di Psicogeriatria - AIP), Brescia, Italy; 9 Italian Society of Gerontology and Geriatrics (Società Italiana di Gerontologia e Geriatria - SIGG), Firenze, Italy; 10 Australian Institute for Suicide Research and Prevention, Griffith University, Nathan, Queensland, Australia; 11 Medicine and Rehabilitation Department, Istituto Clinico S.Anna Hospital, Gruppo San Donato, Brescia, Italy; 12 Acute Geriatrics Unit, Fondazione IRCCS San Gerardo dei Tintori, Monza, Italy; 13 YES group (Young Epidemiologist of the Italian Society of Gerontology and Geriatrics), Florence, Italy

**Keywords:** Geriatric setting, COVID-19, mental health, healthcare workers, psychological profile

## Abstract

**Objectives:**

This study aimed to investigate the psychological impact of the COVID-19 pandemic on healthcare workers (HCWs) in geriatric settings.

**Design:**

Online cross-sectional survey.

**Settings and Participants:**

394 geriatric HCWs in Italy.

**Measurements:**

The survey was developed by a multidisciplinary team and disseminated in April 2022 to the members of two geriatric scientific societies (Italian Society of Geriatrics and Gerontology and Italian Association of Psychogeriatrics). The survey examined the experiences related to the COVID-19 pandemic, as well as psychological burden and support. Work-related anxiety and distress related to the pandemic were studied using the SAVE-9 scale (Stress and Anxiety to Viral Epidemics).

**Results:**

Three hundred sixty-four participants (92.4%) changed their job activity during the pandemic and about half (50.9%) failed to cope with this change, 58 (14.7%) had increased work-related anxiety, and 39 (9.9%) work-related stress levels. Three hundred forty (86.3%) participants reported acute stress reaction symptoms, including irritability, depressed mood, headache, anxiety, and insomnia, and 262 (66.5%) required psychological support, mainly from friends/relatives (57.9%) and/or colleagues (32.5%). Furthermore, 342 participants (86.8%) recognized they would benefit from informal and formal psychological support in case of future similar emergencies.

**Conclusions:**

This study highlights the high psychological burden experienced by geriatric HCWs in Italy during the COVID-19 pandemic and emphasizes the need for supportive interventions.

## Introduction

**T**he CoronaVirus Disease 2019 (COVID-19) outbreak, like previous pandemics (1), led to an increased level of psychological distress among both the general population and healthcare workers (HCWs) (2, 3, 4, 5). Several factors, including an increased workload, the lack of preventive measures and therapeutic protocols, as well as the fear of contracting the infection and transmitting it to relatives, friends, and colleagues have been recognized as major contributors to this condition (6, 7).

A previous study highlighted increased anxiety and depressive symptoms among HCWs working during the COVID-19 pandemic (8). Insomnia and sleep problems, distress, burnout, and post-traumatic stress disorders (PTSDs) have also been reported (9, 10, 11, 12, 13).

HCWs who work in geriatric settings (14, 15), may have experienced an even higher proportion of these disorders, given that their patients (typically affected by frailty and dementia (16, 17)), were the most vulnerable to COVID-19 and the most susceptible to adverse outcomes (18).

Considering the varying degrees of psychological burden experienced by HCWs, it is crucial to identify those who have been most affected by the pandemic's psychological consequences, in order to plan appropriate and targeted interventions (6, 12, 19). With Italy being one of the countries with the highest prevalence of older people and the heaviest pandemic burden (20), we conducted a survey to investigate the personal attitudes and job experiences of geriatric HCWs in our country. Additionally, hypothesizing that female and younger HCWs may be more vulnerable to the psychological burden of COVID-19, we examined potential sex- or age-related differences in this context.

## Methods

### Study Design

We conducted a nationwide survey of HCWs, including physicians, nurses, psychologist, and others, belonging to the Italian Society of Geriatrics and Gerontology (SIGG) and the Italian Association of Psychogeriatrics (AIP).

The questionnaire was developed from January to March 2022 by a multidisciplinary group including geriatricians, geriatric residents, and one psychologist.

The survey was implemented using REDCap, a secure web application for building and managing online surveys and databases (https://projectredcap.org).

The survey was then pre-tested by two experts in the field (AB and GB), who provided feedback on “Face Validity and Content Validity.” Lastly, it was “pilot tested” by all the creators, evaluating duration, flow, relevance, and acceptability, and questions were screened for redundancy, relevance, and clarity.

### Survey Administration

Both scientific societies (SIGG and AIP) invited all their members to complete the survey, which was launched via email in April 2022. Each participant was also invited to transmit the survey to other colleagues and other HCWs working in the geriatric field to increase the response rate and cost-effectiveness of data collection. The survey was open and anonymous.

### Description of the questionnaire

The English version of the questionnaire is reported in Appendix 1. It included 45 questions divided into three different sections. The first section examined the job experience of the participants and investigated the changes in their job position since the COVID-19 pandemic.

The second section investigated their psychological burden with the SAVE-9 scale (Stress and Anxiety to Viral Epidemics - 9 items) (21), a validated tool developed to assess work anxiety and stress responses among HCWs during viral outbreaks. It consists of nine questions with a five-point Likert scale ranging from 0 (never) to 4 (always). The questions are grouped into two subgroups: «Factor I», consisting of questions one through five and eight, and «Factor II», comprising the remaining questions. A cut-off score of 15 is used to identify work-related anxiety within “Factor I”, while a cut-off score of 22 from the overall score of all questions is used to identify stress, based on previous research (22).

In the last section, participants were asked to report whether they had used psychological or pharmacological support to cope with COVID-19-related psychological distress. Furthermore, the participant's perception about: a. the adequacy of the healthcare organizations in dealing with the pandemic's toll; b. the role of the civil society in influencing the HCWs' distress levels; c. the solidarity between colleagues during the pandemic's waves were investigated.

### Statistical Analysis

Only complete questionnaires were included in the final analysis. Quantitative data are presented as mean (standard deviation) or median (interquartile range), and qualitative data as count (percentage). Stratified analyses based on sex, age, and SAVE-9 score were performed using Student t-test, Mann-Whitney-Wilcoxon Test, and Chi-square to explore differences between the groups. In addition, Bonferroni's correction was used to minimize type I errors. Stepwise logistic regression was performed to identify the factors associated with anxiety and/or stress according to the SAVE-9 score.

Analyses were performed in R software, version 4.1.1 (23, 24).

## Results

Four hundred and seventy HCWs completed the survey. Of these, 76 were excluded due to incomplete data, leaving a final sample of 394 participants.

The demographic characteristics of the final sample are shown in Table 1. Since the beginning of the pandemic, most participants (92.4%) underwent a change in their job, mainly related to location, shifts, or, more generally, their habits. Among those who experienced a change, 185 (50.9%) struggled or failed to adapt, while 141 (38.7%) coped positively.

**Table 1:** Main characteristics of the sample (n = 394)

**Characteristic**	**Mean (SD) or N (%)**
Age (years)	44.9 (14.4)
Living alone	61 (15.5)
Occupation	
Physician	319 (81.0)
Nurse	18 (4.6)
Psychologist	38 (9.6)
Other	19 (4.8)
Working area	
Northern	270 (68.5)
Central	60 (15.2)
Southern	38 (9.6)
Insular	26 (6.6)
Length of working experience	
> 1 year	11 (2.8)
1 year to 5 years	120 (30.5)
5 years to 10 years	48 (12.2)
10 years to 20 years	68 (17.3)
> 20 years	147 (37.3)
Variables related to COVID-19 pandemics	
Participants who were working with patients affected by COVID-19	323 (82.0)
Job setting*§	
Community	57 (17.6)
Acute hospital ward	189 (58.5)
Subacute care	80 (24.8)
Emergency Room	27 (8.4)
Other	70 (21.7)
N. of months working with COVID-19 patients	9.7 (6.7)
Participants who got SARS-CoV-2 infection	186 (47.2)
Colleagues/relatives/friends who got COVID-19	
Yes, hospitalized	238 (60.4)
Yes, died	139 (35.3)

SD = standard deviation; * N = 323; § multiple choices were permitted.

Furthermore, 323 participants (82%) worked with patients affected by COVID-19, mainly in acute wards (58.5%), for a mean number of 9.7 (6.7) months. About half of the participants got SARS-CoV-2 infection (47.2%), while 238 (60.4%) reported having friends/relatives and/or colleagues who were hospitalized for COVID-19.

The SAVE-9 scale showed that 14.7% of participants had a score greater than or equal to 15, suggesting the presence of anxious symptoms, whereas 9.9% exhibited a score higher than 22, suggesting work-related stress.

**Figure 1:**
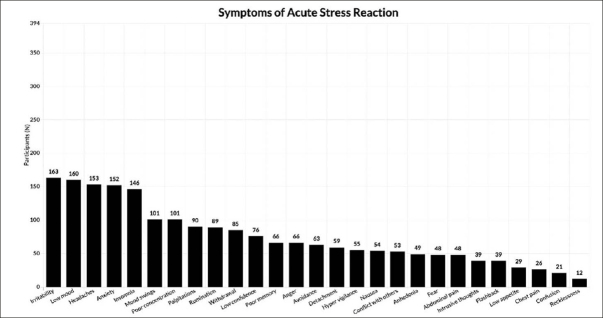
Absolute frequency of symptoms of Acute Stress Reaction

Most participants (86.3%) experienced acute stress reaction symptoms, with a higher prevalence of irritability (41.5%), depressed mood (40.6%), headache (38.8%), anxiety (38.6%), and insomnia (37.1%) (Table 2 and Figure 1).

**Table 2:** Psychological distress assessment (n = 394)

**Characteristic**	**Mean (SD) or N (%)**
SAVE-9 total	13.7 (5.6)
SAVE-9 ≥ 15	58 (14.7)
SAVE-9 ≥ 22	39 (9.9)
Symptoms of acute stress reaction§	5.2 (4.9)
Physical	255 (64.7)
Behavioral	223 (56.6)
Emotional	259 (65.7)
Cognitive	194 (49.2)

SD = standard deviation; SAVE-9 = Stress and Anxiety to Viral Epidemics-9; § multiple choices were permitted.

Around two-thirds of participants needed psychological support during the pandemic. Most relied on friends/relatives (57.9%) and/or colleagues (32.5%), while only 13.2% requested the support of a psychologist and 0.8% of a psychiatrist. Three hundred and forty-two participants (86.8%) admitted they would benefit from psychological support in case of future stressful events, as provided by relatives/friends (55.1%), psychologists (53.8%), colleagues (27.4%), and psychiatrists (5.8%). Most of the participants did not have a psychological support service (39.3%) or were unaware of having it (14.0%) in their workplace (Supplementary Table 1).

Thirty-eight HCWs (9.6%) used medications, mostly antidepressants (81.6%) and benzodiazepines (52.6%), before and during the pandemic.

About half (51%) of participants judged inadequate the response provided by their healthcare organization during the pandemic, and 64.2% perceived a progressive reduction of solidarity among HCWs between the second and third pandemic waves.

Finally, most participants identified the media emphasis on medical uncertainties (62.9%), the “no-vax” initiatives (53.6%), and the catastrophic scenarios depicted by social media (46.4%) as significant factors influencing their psychological distress.

We also examined differences in age, sex, and SAVE-9 scores among participants (Supplementary Tables 2, 3, and 4). Women (258; 65.5%) and younger HCWs (243; 61.7% aged > 50 years) reported experiencing more acute stress reaction symptoms and a greater need for psychological support compared to male and older participants. However, no significant differences related to sex and age were found in their ability to cope with job changes or in their SAVE-9 scores. Additionally, those with SAVE-9 scores indicative of anxiety and/or stress (16.0%) reported a higher frequency of acute stress reaction symptoms and difficulty adapting to pandemic-related work changes, compared to those with scores below the cut-off. No other significant differences were observed. Logistic regression analysis (Supplementary Table 5) revealed that participants from Northern Italy (compared to Insular and Southern Italy) and those with more than 20 years of experience (compared to less than 5 years) were less likely to report a SAVE-9 score indicative of work-related stress or anxiety (OR 0.41, 95% CI 0.20–0.85 and OR 0.11, 95% CI 0.02–0.56, respectively). Moreover, participants who received formal or informal psychological support were more likely to report a SAVE-9 score indicative of work-related stress or anxiety (OR 4.40, 95% CI 1.12–16.0 and OR 2.30, 95% CI 1.15–4.86, respectively) compared to participants who did not receive any support. Additionally, participants who used pharmacological treatment were more likely to report a higher SAVE-9 score than those who did not (OR 2.27, 95% CI 0.98–5.05).

## Discussion

Our study shows that the COVID-19 pandemic has led to changes in job activities for many geriatric healthcare workers (HCWs), and around half of them have had trouble coping with these changes. Moreover, a non-negligible proportion of HCWs have developed symptoms of acute stress reactions and anxiety disorders. Although many HCWs were supported by friends, relatives, and colleagues, only a small percentage received formal psychological support from psychologists and/ or psychiatrists. However, many HCWs expressed the need for structured and formal support in future situations causing psychological distress, such as other emergencies.

Previous studies conducted during the COVID-19 pandemic found a relevant psychological burden among HCWs (15, 25, 26, 27), but only two explored the levels of distress among HCWs working in the geriatric fields. Both studies were conducted in Italy among healthcare professionals of nursing homes or rehabilitation facilities. The first one found a 43% prevalence of moderate-to-severe anxiety and/or post-traumatic symptoms, with an 18% prevalence of multiple conditions (28). The second study found higher resilience and distress levels among physicians, with an increased risk for distress among those who reported low resilience levels (14). However, neither study included those geriatric HCWs working in acute hospitals and community services and evaluated the personal and social resources used to cope with the pandemic.

Our survey is, therefore, the first investigation describing the HCWs' psychological burden related to the COVID-19 pandemic across a heterogeneous and nationwide group of geriatric healthcare professionals.

As well known, the pandemic led to stressful working conditions in several ways. For instance, many HCWs may have been assigned to other (than usual) hospital wards, may have asked to carry out unfamiliar tasks, or could simply have worked under increased emotional pressure. In addition, lack of personal protective equipment and fear of contracting the infection or contaminating family, friends, or colleagues may have contributed to an increased perception of job-related stress (6, 7, 29). In line with this assumption, we found that most HCWs changed their working conditions (i.e., role, staff, or working time) since the beginning of the pandemic, and about half of them failed to deal with it. This probably led to the development of work-related anxiety and acute stress reaction symptoms, with a high prevalence of behavioral and emotional ones. The most reported symptoms were irritability, depressed mood, headache, anxiety, and insomnia. This is in line with a previous systematic review, finding a median frequency of 24% anxious disorders, 21% depressive symptoms, and 37% sleep disorders among HCWs, mainly frontline nurses and physicians (30). Similar results were also obtained by Pappa et al., who reported a pooled 23% prevalence of anxiety, 22% depression, and 34% insomnia (8), and by Riello et al., who found a 22% frequency of anxious disorders (28).

Over time, the negative effects of stress may impact on job conditions, family, and other social relationships. Therefore, it is crucial to support the mental well-being of HCWs with specific interventions, such as changing routines, and providing personal protective equipment and psychological support (4).

Our data are consistent with the literature about informal psychological support to HCWs (7, 30, 31, 32, 33). Cai et al. found that seeking help from family and friends was an important supportive measure (31), whereas counseling a psychologist was not. Similar findings were obtained in a study by De Leo et al. (33). In our study, we found that HCWs frequently relied on their relatives and friends to cope with COVID-19 stress, while only a minority of them relied on a psychologist or a psychiatrist. HCWs can likely benefit from professional mental health interventions more than they believed; the under-recognition of this need could be related to their occupational culture or the fear of being perceived as unsteady (34). However, it is noteworthy that most participants recognized they would rely on psychological support in case of future distressful events, such as a new health emergency. Interestingly, we found that participants who sought either formal or informal psychological support or used pharmacological treatments were more likely to have higher SAVE-9 scores compared to those who did not receive any form of support or treatment. This may suggest that those who actively sought support might have experienced more prominent symptoms of work-related stress and anxiety or a heightened awareness of their mental health needs. However, it is important to emphasize that this association does not establish a causal relationship. Further research is necessary to explore the underlying reasons and potential effects of seeking support on stress and anxiety levels among HCWs.

Another aspect that could positively contribute to reducing the psychological burden of HCWs is a supportive community (7, 31, 35). This issue holds significant relevance, as the majority of participants acknowledged that certain attitudes within civil society might have contributed to an increase in their stress levels. This recognition underscores the importance of implementing strategies aimed at fostering a healthy work environment and supportive community.

However, it must be considered that not all the staff members developed distress to the same degree (6). It may therefore be appropriate to identify the vulnerable individuals within the category of HCWs and give them psychological support. According to recent meta-analyses, being a frontline staff, a nurse, a woman, or having younger age were risk factors for adverse mental health outcomes during the COVID-19 pandemic (3, 7, 8).

In agreement with these findings, the two Italian studies conducted in long-term geriatric facilities showed that females had more severe symptoms than males and higher anxious disorders (28) or psychological distress (14). Unexpectedly, we failed to identify significant sex-related differences in job changes and adaptation or in work-related anxiety and stress levels. However, women reported higher acute stress reaction symptoms than men and a greater need for psychological support. It is recognized that women are more vulnerable than men to stress and anxiety after a trauma, such as a pandemic (8, 36). Moreover, the difference in the assessment tools and in the cut-off scores used by the surveys might lead to heterogeneity in the study results (8).

When taking age into consideration, we found that younger and older HCWs dealt similarly with the changes in their job activity. Moreover, on average, younger HCWs reported higher SAVE-9 values, although there were no significant differences in anxiety and stress subscores between the two age groups. However, younger HCWs reported an increase in acute stress reaction symptoms and a need for both informal and professional support. These results are consistent with the current evidence that identifies young age as a risk factor for psychological distress (7, 11, 19, 29). Additionally, our study revealed that participants with more than 20 years of work experience were less likely to report a higher SAVE-9 score compared to participants with less than 5 years of experience. This suggests that not only age but also work experience may contribute to the ability to cope with stressors in the healthcare environment. However, further investigation is needed to determine the factors underlying this relationship and the effectiveness of targeted interventions to promote mental health in HCWs, especially during and after emergency situations. Future studies should prioritize identifying specific factors associated with elevated distress levels across different age groups of HCWs. Additionally, it is crucial to determine the types of support that can effectively address these factors and alleviate distress.

### Strengths and limitations

The main strength of this study is its nationwide extension and, thus, its potential representativeness of the Italian geriatric HCWs' psychological burden. However, some biases must be acknowledged. First, the online sampling technique and the channels used to disseminate the survey could have introduced a selection bias. Moreover, due to the dissemination strategy, the response rate is hard to estimate. Indeed, we must also consider that we have no data regarding those who refused to participate in the survey, which may have differed from participants concerning demographic, socioeconomic, cultural, lifestyle and health status (37).

## Conclusions

This survey underlines a high psychological burden related to the COVID-19 pandemic among geriatric HCWs and the consequent need for supportive interventions. These interventions should aim to strengthening the health organizational structure and empowering HCWs through specific approaches and professional support programs.

Further studies are needed to investigate better the factors related to HCWs' mental outcomes in emergency situations, to plan preventive and supportive strategies.

## Electronic supplementary material

Appendix 1. Survey content, approximately 60 KB.

*Author's contributions:* Alice M. Ornago, Elena Pinardi, Alberto Zucchelli, Caterina Trevisan, Maddalena Volterrani, Stefano Cacciatore, and Chiara Ceolin contributed to the study conception and design and to acquisition of data; data analysis was performed by Alice M. Ornago, Alberto Zucchelli, and Elena Pinardi. Data interpretation was provided by all the authors. The first draft of the manuscript was written by Alice M. Ornago and Giuseppe Bellelli and all authors revised the manuscript. All authors approved the final manuscript's version.

*Funding:* The study received no funding. Open access funding provided by Università degli Studi di Milano - Bicocca within the CRUI-CARE Agreement.

*Ethical consideration:* This research complies with the ethical rules for human experimentation stated in the Declaration of Helsinki.
