# Supplementary material for: Mental Health among Geriatric Healthcare Workers in Italy during the COVID-19 Pandemic: Results from a National Survey
Source: J Nutr Health Aging. 2023 Aug 11;27(8):626–31. doi: 10.1007/s12603-023-1958-1 (PMC12877578; doi:10.1007/s12603-023-1958-1)
Supplement: Supplementary file 1 — Appendix 1. Survey content, approximately 60 KB. [file mmc1.docx]

**Appendix 1. Survey content**

1. How old are you? ______
2. Gender:
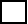
 Male
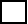
 Female
3. Who do you live with?
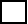
 Alone
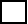
 In company
4. In which region do you work?
5. Abruzzo
6. Basilicata
7. Calabria
8. Campania
9. Emilia-Romagna
10. Friuli-Venezia-Giulia
11. Lazio
12. Liguria
13. Lombardia
14. Marche
15. Molise
16. Piemonte
17. Puglia
18. Sardegna
19. Sicilia
20. Toscana
21. Trentino-Alto Adige
22. Umbria
23. Valle d’Aosta
24. Veneto
25. How many years have you been working?
26. Less than a year
27. From one to five years
28. From five to ten years
29. From ten to twenty years
30. More than twenty years
31. What is your main job?
32. Hospital Physician
33. Physician in nursing or long-term care facilities
34. Physician in rehabilitation facilities
35. Physician in Hospice
36. General Practitioner
37. Physician in Community services
38. Resident
39. Hospital nurse
40. Nurse in nursing or long-term care facilities
41. Nurse in rehabilitation facilities
42. Nurse on territory
43. Psychologist
44. Other
45. There has been a job change over the past two years?
46. None
47. Yes, of role
48. Yes, of setting
49. Yes, of working team
50. Yes, of shift
51. Yes, other (specify): ___________
52. Have you had the perception that your work habits have changed?
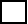
 Yes
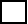
 No
53. How did you experience this change?
54. I was unable to adapt
55. I struggled to adapt
56. I wasn’t affected by the change
57. I adapted positively
58. I benefited from the change
59. In which periods did you work in contact with patients affected by COVID-19?
60. I have never worked with patients affected by COVID-19
61. Between January and April 2020
62. Between May and August 2020
63. Between September and December 2020
64. Between January and April 2021
65. Between May and August 2021
66. Between September and December 2021
67. Between January and March 2022
68. If you worked with patients affected by COVID-19, in which setting?
    1. On the territory
    2. Acute care settings
    3. Subacute care settings
    4. Emergency room settings
    5. Other settings
69. For how many months, in the last two years, have you worked with patients affected by COVID-19? _______________
70. Have you ever tested positive for COVID-19?
71. No, I never tested positive for COVID-19
72. Yes, between January and April 2020
73. Yes, between May and August 2020
74. Yes, between September and December 2020
75. Yes, between January and April 2021
76. Yes, between May and August 2021
77. Yes, between September and December 2021
78. Yes, between January and March 2022
79. Did you have any symptoms the first time you tested positive for COVID-19?
80. No
81. Yes, treated at home
82. Yes, treated in hospital
83. Yes, treated in ICU
84. Did you have any symptoms the second time you tested positive for COVID-19?
85. No
86. Yes, treated at home
87. Yes, treated in hospital
88. Yes, treated in ICU
89. Did you have any symptoms the third time you tested positive for COVID-19?
90. No
91. Yes, treated at home
92. Yes, treated in hospital
93. Yes, treated in ICU
94. Have you had relatives/friends or colleagues hospitalized for COVID-19?
95. Nobody
96. Yes, colleagues
97. Yes, relatives/friends
98. Yes, both colleagues and relatives/friends
99. If yes, when?
100. Between January and April 2020
101. Between May and August 2020
102. Between September and December 2020
103. Between January and April 2021
104. Between May and August 2021
105. Between September and December 2021
106. Between January and March 2022
107. Have you had relatives/friends or colleagues who died for COVID-19?
108. Nobody
109. Yes, colleagues
110. Yes, relatives/friends
111. Yes, both colleagues and relatives/friends
112. If yes, when?
113. Between January and April 2020
114. Between May and August 2020
115. Between September and December 2020
116. Between January and April 2021
117. Between May and August 2021
118. Between September and December 2021
119. Between January and March 2022
120. Stress and Anxiety to Viral Epidemic – 9 items (SAVE-9) for healthcare workers.

|  | Never | Rarely | Sometimes | Often | Always |
| --- | --- | --- | --- | --- | --- |
| Are you afraid the virus outbreak will continue indefinitely? | 0 | 1 | 2 | 3 | 4 |
| Are you afraid your health will worsen because of the virus? | 0 | 1 | 2 | 3 | 4 |
| Are you worried that you might get infected? | 0 | 1 | 2 | 3 | 4 |
| Are you more sensitive towards minor physical symptoms than usual? | 0 | 1 | 2 | 3 | 4 |
| Are you worried that others might avoid you even after the infection risk has been minimized? | 0 | 1 | 2 | 3 | 4 |
| Do you feel skeptical about your job after going through this experience? | 0 | 1 | 2 | 3 | 4 |
| After this experience, do you think you will avoid treating patients with viral illnesses? | 0 | 1 | 2 | 3 | 4 |
| Do you worry your family or friends may become infected because of you? | 0 | 1 | 2 | 3 | 4 |
| Do you think that your colleagues would have more work to do due to your absence from a possible quarantine and might blame you? | 0 | 1 | 2 | 3 | 4 |

1. Have you experienced any of the following signs or symptoms in the last month?
2. Palpitations
3. Nausea
4. Low appetite
5. Chest pain
6. Headaches
7. Abdominal pains
8. Insomnia
9. Avoidance
10. Recklessness
11. Detachment
12. Withdrawal
13. Irritability
14. Conflict with others
15. Anxiety
16. Low mood
17. Anger
18. Fear
19. Mood swings
20. Anhedonia
21. Low confidence
22. Poor concentration
23. Intrusive thoughts
24. Flashbacks
25. Poor memory
26. Confusion
27. Hyper vigilance
28. Rumination
29. In your life journey, to cope with symptoms of psychological discomfort, have you used psychological support?
30. No
31. Yes, before the COVID-19 pandemic
32. Yes, during the COVID-19 pandemic
33. In your life journey, to cope with symptoms of psychological discomfort, have you used specific pharmacological treatment for at least 2 continuous months?
34. No
35. Yes, before the COVID-19 pandemic
36. Yes, during the COVID-19 pandemic
37. If yes, which of the following drugs have you used?
38. Benzodiazepines
39. Hypnotics
40. Antidepressants
41. Antipsychotics
42. Others (specify): ___________
43. How have you dealt with any psychological distress in the past two years?
    1. I didn't need psychological support
    2. With the help of a psychologist
    3. With the help of a psychiatrist
    4. With the help of relatives/friends
    5. With the help of colleagues
44. How would you like to deal with any future psychological distress?
    1. I don't think I will need support
    2. With the help of a psychologist
    3. With the help of a psychiatrist
    4. With the help of relatives/friends
    5. With the help of colleagues
45. Are there psychological support services for staff in your work setting?
46. None
47. Yes, since before the COVID-19 pandemic
48. Yes, since before the COVID-19 pandemic but reinforced during the last two years
49. Yes, established during the COVID-19 pandemic
50. I’m not aware
51. If yes, what kind of services are available?
52. Psychological interview
53. Telephone support service
54. Self-help courses
55. Other (specify): ________
56. Do you think that in your workplace there has been an adequate health organizational response to deal with the COVID-19 pandemic?
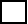
 Yes
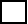
 No
57. Do you think that society attitudes have negatively affected your level of work stress?
58. No
59. Yes, negative attitudes on social
60. Yes, the press's overemphasis on potential errors in health care
61. Yes, the no-vax attacks
62. Yes, other (specify): __________
63. Do you think there has been a difference in terms of professional solidarity between the various pandemic waves?
64. No
65. Yes, with an increasing trend
66. Yes, with a decreasing trend

**Supplementary Materials**

**Supplementary Table 1. Focus on psychological support**

| **Characteristic** | **Mean (SD) *or* N (%)** | |
| --- | --- | --- |
| Need for psychological support  Never  Before the pandemic  During the pandemic | 264 (67.0)  103 (26.1)  51 (12.9) | |
| Need for pharmacological treatment  Never  Before the pandemic  During the pandemic | 356 (90.4)  28 (7.1)  19 (4.8) | |
| Type of pharmacological treatment*^§^  Benzodiazepine  Hypnotic  Antidepressant  Antipsychotic  Other | 20 (52.6)  4 (10.5)  31 (81.6)  4 (10.5)  3 (7.9) | |
| Psychological support for the last two years^§^  None  Psychologist  Psychiatrist  Relatives/friends  Colleagues | | 132 (33.5)  52 (13.2)  3 (0.8)  228 (57.9)  128 (32.5) |
| Psychological support for future distress situations^§^  None  Psychologist  Psychiatrist  Relatives/friends  Colleagues | | 52 (13.2)  212 (53.8)  23 (5.8)  217 (55.1)  108 (27.4) |
| Support services available at workplace  None  Since before the pandemic  Reinforced during the pandemic  Established during the pandemic  Not aware | | 155 (39.3)  74 (18.8)  46 (11.7)  64 (16.2)  55 (14.0) |
| Type of support services available at workplace**^§^  Psychological interview  Telephone support service  Self-help courses  Other | | 175 (95.1)  40 (21.7)  11 (6.0)  4 (2.2) |

SD = standard deviation; * N = 38; ** N = 184; ^§^ multiple choices were permitted.

**Supplementary Table 2. Analyses stratified by sex**

| Characteristic | Men | Women | p-value | p-adjusted |
| --- | --- | --- | --- | --- |
| *n* | 136 | 258 |  |  |
| Age  < 50 years  ≥ 50 years | 51.8 (15.1)  59 (43.4)  77 (56.6) | 41.2 (12.7)  184 (71.3)  74 (28.7) | **< 0.001** | **< 0.001** |
| Live alone | 21 (15.4) | 40 (15.5) | 1 | 1 |
| Occupation  Physician  Nurse  Psychologist  Other | 123 (90.4)  3 (2.2)  5 (3.7)  5 (3.7) | 196 (76.0)  15 (5.8)  33 (12.8)  14 (5.4) | **< 0.001** | 0.057 |
| Length of working experience  < 1 year  1 year to 5 years  5 years to 10 years  10 years to 20 years  > 20 years | 5 (3.7)  26 (19.1)  6 (4.4)  23 (16.9)  76 (55.9) | 6 (2.3)  94 (36.4)  42 (16.3)  45 (17.4)  71 (27.5) | **< 0.001** | **< 0.001** |
| Job changes in the last two years^†§^  None | 31 (22.8) | 44 (17.1) | 0.179 | 1 |
| Change of role  Change of setting  Change of team  Change of shift  Other | 39 (28.7)  65 (47.8)  41 (30.1)  48 (35.3)  7 (5.1) | 78 (30.2)  127 (49.2)  99 (38.4)  112 (43.4)  19 (7.4) |  |  |
| Perception of change in the working habits | 113 (83.1) | 221 (85.7) | 0.556 | 1 |
| Adaptation to job change*  Unable to adapt  Struggled to adapt  Not affected by the change  Positively adapted  Benefited from the change | 3 (2.4)  60 (47.2)  14 (11.0)  35 (27.6)  15 (11.8) | 6 (2.5)  116 (48.9)  24 (10.1)  71 (30)  20 (8.4) | 0.852 | 1 |
| Participants who were working with patients affected by  COVID-19 | 111 (81.6) | 212 (82.2) | 0.891 | 1 |
| Setting of work**^§^  Territorial healthcare  Acute ward  Subacute care  Emergency Room  Other | 18 (16.2)  64 (57.7)  28 (25.2)  7 (6.3)  20 (18.0) | 39 (18.4)  125 (59.0)  52 (24.5)  20 (9.4)  50 (23.6) |  |  |
| N. months working with COVID-19 patients | 11.1 (7.6) | 9 (6.0) | **0.011** | 0.199 |
| Participants who got SARS-CoV-2 infection | 66 (48.5) | 120 (46.5) | 0.751 | 1 |
| Colleagues/relatives/friends who got COVID-19  Yes, hospitalized  Yes, died | 99 (72.8)  61 (44.9) | 139 (53.9)  78 (30.2) | **0.001**  **<0.001** | **0.007**  0.098 |
| Psychological distress assessment |  |  |  |  |
| SAVE-9 total | 12.9 (5.4) | 14 (5.6) | 0.060 | 1 |
| SAVE-9 ≥ 15 | 19 (14.0) | 39 (15.1) | 0.876 | 1 |
| SAVE-9 ≥ 22 | 9 (6.6) | 30 (11.6) | 0.154 | 1 |
| Symptoms of acute stress reaction | 5.6 (4.9) | 4.3 (4.7) | **<0.001** | **0.014** |
| Psychological support for the last two years^†§^  None | 66 (48.5) | 66 (25.6) | **< 0.001** | **< 0.001** |
| Psychologist  Psychiatrist  Friends/relatives  Colleagues | 9 (6.6)  1 (0.7)  63 (46.3)  26 (19.1) | 43 (16.7)  2 (0.8)  165 (64)  102 (39.5) |  |  |
| Psychological support for future distress situations^†§^  None | 27 (19.9) | 25 (9.7) | **0.007** | 0.133 |
| Psychologist  Psychiatrist  Friends/relatives  Colleagues | 53 (39)  7 (5.1)  76 (55.9)  29 (21.3) | 159 (61.6)  16 (6.2)  141 (54.7)  79 (30.6) |  |  |
| Social attitudes affecting stress levels^†§^  None | 21 (15.4) | 50 (19.4) | 0.408 | 1 |
| Social medias scenarios  Media emphasis on medical uncertainties  No-vax” initiatives  Other attitudes | 70 (51.5)  87 (64)  76 (55.9)  8 (5.9) | 113 (43.8)  161 (62.4)  135 (52.3)  19 (7.4) |  |  |

Data are shown as N (%) or mean (DS); DS = standard deviation; SAVE-9 = Stress and Anxiety to Viral Epidemics-9; **^†^** p-value for “none” vs. “other answer”; * N_men_= 127; N_women_ = 237; ** N_men_ = 111; N_women_ = 212; ^§^ multiple choices were permitted.

**Supplementary Table 3. Analyses stratified by age**

| Characteristic | Age < 50 y | Age ≥ 50 y | p-value | p-adjusted |
| --- | --- | --- | --- | --- |
| *n* | 243 | 151 |  |  |
| Male | 59 (24.3) | 77 (51.0) | **< 0.001** | **< 0.001** |
| Age | 34.9 (7) | 61 (6.7) | **<0.001** | **< 0.001** |
| Live alone | 42 (17.3) | 19 (12.6) | 0.252 | 1 |
| Occupation  Physician  Nurse  Psychologist  Other | 194 (79.8)  13 (5.3)  28 (11.5)  8 (3.3) | 125 (82.8)  5 (3.3)  10 (6.6)  11 (7.3) | **0.096** | **1** |
| Length of working experience  < 1 year  1 year to 5 years  5 years to 10 years  10 years to 20 years  > 20 years | 11 (4.5)  120 (49.4)  46 (18.9)  56 (23.0)  10 (4.1) | -  -  2 (1.3)  12 (7.9)  137 (90.7) | **< 0.001** | **0.009** |
| Job changes in the last two years^†§^  None | 29 (11.9) | 46 (30.5) | **<0.001** | **< 0.001** |
| Change of role  Change of setting  Change of team  Change of shift  Other | 84 (34.6)  136 (56.0)  109 (44.9)  114 (46.9)  19 (7.8) | 33 (21.9)  56 (37.1)  31 (20.5)  46 (30.5)  7 (4.6) |  |  |
| Perception of change in the working habits | 211 (86.8) | 123 (81.5) | 0.152 | 1 |
| Adaptation to job change*  Unable to adapt  Struggled to adapt  Not affected by the change  Positively adapted  Benefited from the change | 7 (3.1)  115 (50.4)  24 (10.5)  66 (28.9)  16 (7.0) | 2 (1.5)  61 (44.9)  14 (10.3)  40 (29.4)  19 (14.0) | 0.237 | 1 |
| Participants who were working with patients affected by  COVID-19 | 212 (87.2) | 111 (73.5) | **0.001** | **0.017** |
| Setting of work**^§^  Territorial healthcare  Acute ward  Subacute care  Emergency Room  Other | 33 (15.6)  140 (66.0)  55 (25.9)  23 (10.8)  36 (17.0) | 24 (21.6)  49 (44.1)  25 (22.5)  4 (3.6)  34 (30.6) |  |  |
| N. months of working with COVID-19 patients | 9.1 (6.1) | 10.9 (7.5) | **< 0.001** | **< 0.001** |
| Participants who got SARS-CoV-2 infection | 124 (51.0) | 62 (41.1) | 0.061 | 1 |
| Colleagues/relatives/friends who got COVID-19  Yes, hospitalized  Yes, died | 127 (52.3)  68 (28.0) | 111 (73.5)  71 (47.0) | **<0.001**  **<0.001** | **< 0.001**  **< 0.001** |
| Psychological distress assessment |  |  |  |  |
| SAVE-9 total | 14.4 (5.5) | 12.5 (5.4) | **<0.001** | **0.015** |
| SAVE-9 ≥ 15 | 41 (16.9) | 17 (11.3) | 0.166 | 1 |
| SAVE-9 ≥ 22 | 30 (12.3) | 9 (6.0) | 0.055 | 1 |
| Symptoms of acute stress reaction | 6.2 (5) | 3.6 (4.3) | **<0.001** | **< 0.001** |
| Psychological support for the last two years^†§^  None | 66 (27.2) | 66 (43.7) | **0.001** | **0.020** |
| Psychologist  Psychiatrist  Friends/relatives  Colleagues | 45 (18.5)  2 (0.8)  153 (63.0)  88 (36.2) | 7 (4.6)  1 (0.7)  75 (49.7)  40 (26.5) |  |  |
| Psychological support for future distress situations^†§^  None | 18 (7.4) | 34 (22.5) | **< 0.001** | **< 0.001** |
| Psychologist  Psychiatrist  Friends/relatives  Colleagues | 159 (65.4)  18 (7.4)  144 (59.3)  73 (30.0) | 53 (35.1)  5 (3.3)  73 (48.3)  35 (23.2) |  |  |
| Social attitudes affecting stress levels^†§^  None | 41 (16.9) | 30 (19.9) | 0.500 | 1 |
| Social media scenarios  Media emphasis on medical uncertainties  No-vax” initiatives  Other attitudes | 114 (46.9)  157 (64.6)  143 (58.8)  21 (8.6) | 69 (45.7)  91 (60.3)  68 (45)  6 (4.0) |  |  |

Data are shown as N (%) or mean (DS); DS = standard deviation; y = years; SAVE-9 = Stress and Anxiety to Viral Epidemics-9; **^†^** p-value for “none” vs. “other answer”; * N_under_ = 228; N_over_ = 136; **N_under_ = 212; N_over_ = 111; ^§^ multiple choices were permitted.

**Supplementary Table 4. Analyses stratified by SAVE-9 score**

| Characteristic | SAVE-9  < 15 | SAVE-9 ≥ 15 *and/or* ≥ 22 | p-value | p-adjusted |
| --- | --- | --- | --- | --- |
| *n* | 331 | 63 |  |  |
| Male | 116 (35.0) | 20 (31.7) | 0.719 | 1 |
| Age | 45.0  [31;58.5] | 36  [23;53] | 0.425 | 1 |
| Live alone | 50 (15.1) | 11 (17.5) | 0.777 | 1 |
| Occupation  Physician  Nurse  Psychologist  Other | 264 (79.8)  17 (5.1)  34 (10.3)  16 (4.8) | 55 (87.3)  1 (1.6)  4 (6.3)  3 (4.8) | 0.444 | 1 |
| Working area  Northern  Central  Southern  Insular | 232 (70.1)  51 (15.4)  25 (7.6)  23 (6.9) | 38 (60.3)  9 (14.3)  13 (20.6)  3 (4.8) | **0.014** | 0.308 |
| Length of working experience  < 1 year  1 year to 5 years  5 years to 10 years  10 years to 20 years  > 20 years | 7 (2.1)  97 (29.3)  38 (11.5)  58 (17.5)  131 (39.6) | 4 (6.3)  23 (36.5)  10 (15.9)  10 (15.9)  16 (25.4) | 0.089 | 1 |
| Job changes in the last two years^†§^  None | 66 (19.9) | 9 (14.3) | 0.383 | 1 |
| Change of role  Change of setting  Change of team  Change of shift  Other | 101 (30.5)  156 (47.1)  114 (34.4)  124 (37.5)  22 (6.6) | 16 (25.4)  36 (57.1)  26 (41.3)  36 (57.1)  4 (6.3) |  |  |
| Perception of change in the working habits | 277 (83.7) | 57 (90.5) | 0.237 | 1 |
| Adaptation to job change*  Unable to adapt  Struggled to adapt  Not affected by the change  Positively adapted  Benefited from the change | 6 (1.8)  134 (40.5)  34 (10.3)  95 (28.7)  34 (10.3) | 3 (4.8)  42 (66.7)  4 (6.3)  11 (17.5)  1 (1.6) | **0.001** | **0.022** |
| Participants who were working with patients affected by COVID-19 | 270 (81.6) | 53 (84.1) | 0.760 | 1 |
| Setting of work**^§^  Territorial healthcare  Acute ward  Subacute care  Emergency Room  Other | 43 (13.0)  163 (49.2)  64 (19.3)  19 (5.7) 61 (18.4) | 14 (22.2)  26 (41.3)  16 (25.4)  8 (12.7)  79(14.3) |  |  |
| N. months of working with COVID-19 patients | 8.0  [4.0;12.0] | 10.0  [4.0;18;0] | 0.183 | 1 |
| Participants who got SARS-CoV-2 infection | 149 (45.0) | 37 (58.7) | 0.063 | 1 |
| Colleagues/relatives/friends who got COVID-19  Yes, hospitalized  Yes, died | 203 (61.3)  115 (34.7) | 35 (55.6)  24 (38.1) | 0.473  0.714 | 1  1 |
| Psychological distress assessment |  |  |  |  |
| SAVE-9 total | 12.0 (4.2) | 22.3 (3.6) | **< 0.001** | **<0.001** |
| Symptoms of acute stress reaction^§^ | 4.0  [1.0;6.0] | 7.0  [4.0;14.0] | **< 0.001** | **< 0.001** |
| Physical  Behavioral  Emotional  Cognitive | 203 (61.3)  174 (52.6)  206 (62.2)  144 (43.5) | 52 (82.5)  49 (77.8)  53 (84.1)  50 (79.4) | **0.002**  **< 0.001**  **0.001**  **< 0.001** | **0.044**  **0.008**  **0.022**  **< 0.001** |
| Psychological support for the last two years^†§^  None | 119 (36.0) | 13 (20.6) | **0.027** | 0.594 |
| Psychologist  Psychiatrist  Friends/relatives  Colleagues | 40 (12.1)  2 (0.6)  187 (56.5)  106 (32.0) | 12 (19.0)  1 (1.6)  41 (65.1)  22 (34.9) |  |  |
| Psychological support for future distress situations^†§^  None | 46 (13.9) | 6 (9.5) | 0.461 | 1 |
| Psychologist  Psychiatrist  Friends/relatives  Colleagues | 175 (52.9)  16 (4.8)  177 (53.5)  86 (26.0) | 37 (58.7)  7 (11.1)  40 (63.5)  22 (34.9) |  |  |
| Social attitudes affecting stress levels ^†§^  None | 63 (19.0) | 8 (12.7) | 0.285 | 1 |
| Social media scenarios  Media emphasis on medical uncertainties  No-vax” initiatives  Other attitudes | 151 (45.6)  200 (60.4)  172 (52.0)  20 (6.0) | 32 (50.8)  48 (76.2)  39 (61.9)  7 (11.1) |  |  |

Data are shown as N (%) or mean (DS) or median [IQR]; DS = standard deviation; IQR = interquartile range; SAVE-9 = Stress and Anxiety to Viral Epidemics-9; **^†^** p-value for “none” vs. “other answer”; * N_<15_= 303; N_≥15/22_ = 61; **N_<15_= 270; N_≥15/22_ = 53; ^§^ multiple choices were permitted.

**Supplementary Table 5. Stepwise Logistic Regression.**

Results of a multivariate logistic regression of psychological burden detected using the SAVE-9 scores.

| **Independent variables** | **Odds Ratio** | **95% CI** | | **p-value** |
| --- | --- | --- | --- | --- |
|  |  | LL | UL |  |
| Age | 1.05 | 1.00 | 1.11 | .032 |
| Working area  Insular and Southern Italy | (Ref.) | (Ref.) | (Ref.) | (Ref.) |
| Central Italy | .47 | .175 | 1.21 | .126 |
| Northern Italy | .41 | .203 | .854 | .015 |
| Length of work experience  < 5 years | (Ref.) | (Ref.) | (Ref.) | (Ref.) |
| 5 years to 20 years | .45 | .177 | 1.10 | .087 |
| > 20 years | .11 | .019 | .556 | .009 |
| SARS-CoV2 infection  No  Yes | (Ref.)  .59 | (Ref.)  .331 | (Ref.)  1.05 | (Ref.)  .075 |
| Pharmacological treatment  No  Yes | (Ref.)  2.27 | (Ref.)  .976 | (Ref.)  5.05 | (Ref.)  .049 |
| Psychological support  None | (Ref.) | (Ref.) | (Ref.) | (Ref.) |
| Formal support | 4.40 | 1.12 | 16.0 | .026 |
| Informal support | 2.30 | 1.15 | 4.86 | .023 |
| Both formal and informal support | 2.04 | .642 | 6.08 | .210 |

Note: The dependent variable is *SAVE-9 burden* coded that 0 = score less than 15 and 1 = score ≥ 15 and/or 22.

CI = confidence interval; LL = lower limit; UL = upper limit.
